# Cleft, Crevice, or the Inner Thigh: ‘Another Place’ for the Establishment of the Invasive Barnacle *Austrominius modestus* (Darwin, 1854)

**DOI:** 10.1371/journal.pone.0048863

**Published:** 2012-11-07

**Authors:** Sally A. Bracewell, Matthew Spencer, Rob H. Marrs, Matthew Iles, Leonie A. Robinson

**Affiliations:** School of Environmental Sciences, University of Liverpool, Liverpool, Merseyside, United Kingdom; National Institute of Water & Atmospheric Research, New Zealand

## Abstract

The proliferation of anthropogenic infrastructure in the marine environment has aided the establishment and spread of invasive species. These structures can create novel habitats in areas normally characterised as void of suitable settlement sites. The habitat requirements of the invasive acorn barnacle *Austrominius modestus* (Darwin, 1854) were assessed using a novel sampling site at Crosby Beach, Liverpool. *Austrominius modestus* has spread rapidly around the UK since its initial introduction, becoming locally dominant in many estuarine areas including the Antony Gormley art installation, ‘Another Place’, at Crosby Beach. The installation consists of 100 replicate solid cast-iron life-size human figures, located at a range of heights on the shore. We recorded the distribution and abundance of *A. modestus* present on all of the statues at various positions during the summer of 2006. The positions varied in location, exposure, direction, and rugosity. Although parameters such as rugosity and exposure did influence patterns of recruitment, they were less important than interactions between shore height and direction, and specific location on the beach. The addition of a suitable substrate to a sheltered and estuarine region of Liverpool Bay has facilitated the establishment of *A. modestus*. Understanding the habitat requirements of invasive species is important if we are to make predictions about their spread and the likelihood of invasion success. *Austrominius modestus* has already become locally dominant in some regions of the UK and, with projections of favourable warming conditions and the global expansion of artificial structures, the continued spread of this species can be expected. The implications of this on the balance between native and invasive species dominance should be considered.

## Introduction

Alien, non-native, introduced, or non-indigenous species (NIS) have become commonplace across many of the worlds ecosystems [1]. If a NIS is able to establish in a region distant from its native range and maintain self-sustaining populations in large numbers it is generally regarded as invasive, elevating to pest status once it has caused significant ecological or economic damage [2]. Successful invasions are contingent on numerous factors (e.g. suitability of habitat [3], interactions with native species [4]), and it has been suggested that approximately only 10% of any established introductions of NIS will become invasive [5], although this value is difficult to quantify [6]. Nevertheless, the number of biological invasions recorded continues to increase [7], [8] and the implications of such increases for levels of biodiversity and ecosystem functioning are now widely acknowledged [9], [10], causing concern amongst conservation biologists and resource managers.

Why some introductions of NIS result in invasions, whilst others do not, is a key question in invasion ecology, the answer to which is multi-faceted and dependent on traits related to both the species in question [11] and those related to the recipient habitat [12]. Greater dispersal abilities, faster growth rates and generation times (i.e. increased propagule pressure) [13], higher stress tolerances [14] and greater capacities for evolutionary change due to higher phenotypic plasticity [15] have emerged as traits related to invasiveness. Likewise, it has been hypothesised that certain types of habitats are more susceptible to invasions than others, with factors such as disturbance [16], [17] and levels of native species diversity [12] being influential.

Recent studies indicate that artificial structures such as piers, pilings, seawalls and other sea defences are particularly vulnerable to invasion by non-native species; however, their contribution as drivers of ecological change has received limited attention [18]. These structures are often located in disturbed habitats, such as ports and estuaries, areas characterised by high shipping traffic and thus an increased abundance of NIS [19], [20]. Artificial structures can create novel environmental conditions (e.g. vertical surfaces, lack of microhabitats), and do not closely resemble native habitats [18]. As such, they are often characterised by low native species diversity [21], and have been found to support assemblages of organisms that greatly contrast those of nearby natural sites [22]. In addition, if NIS are able to become naturalized on these structures, they can act as supplementary recruitment sites that may aid their spread, effectively acting as stepping-stones in areas of otherwise unsuitable habitat [23]. With the rising concern over the consequences of global climate change, the number of artificial structures is likely to increase in the hope that they will provide the necessary protection against such threats as sea level rise and increased storm activity [18]. The implications of this for the management of invasive species should be considered carefully [24], [25].

Understanding the factors that contribute to, or inhibit, the establishment of invasive species is critical for developing effective management techniques and predicting future range expansions [26]. Studies monitoring abundance patterns of NIS can provide valuable information about their distribution and rate of spread [27], [28]. Sessile marine invertebrates are common fouling organisms on artificial structures and their abundance and distribution on these structures could offer insights into the habitat requirements of these species, which in turn could help assess the likelihood of establishment in novel locations and on novel structures. Although patterns in abundance of marine invertebrates with a pelagic larval stage are inextricably linked to variations in larval supply, factors relating to the abiotic features of the recipient habitat or structure are also important in shaping the distribution of organisms [29].

**Figure 1:**
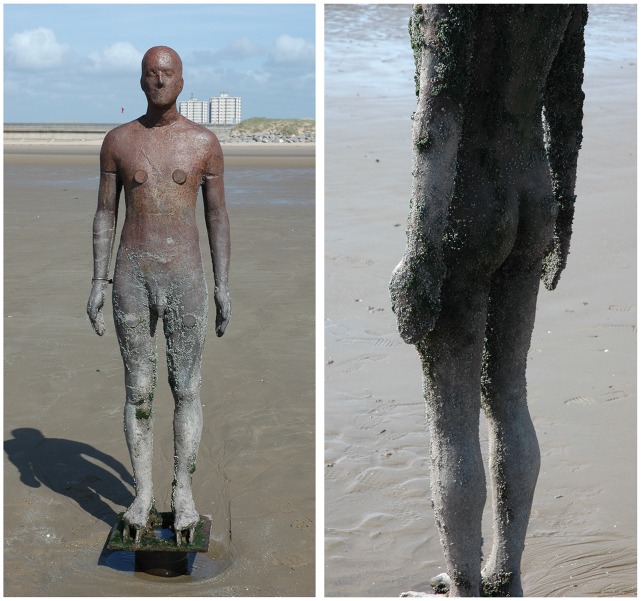
Examples of the life-size cast-iron statues at Crosby Beach, Liverpool. Images show two of the 100 statues that form the art installation ‘Another Place’; one at the higher end of shore height sampled (left) and one at a lower shore height (right). The statues stretch over approximately 3 km of the foreshore and are distributed at a range of tidal heights.

**Figure 2:**
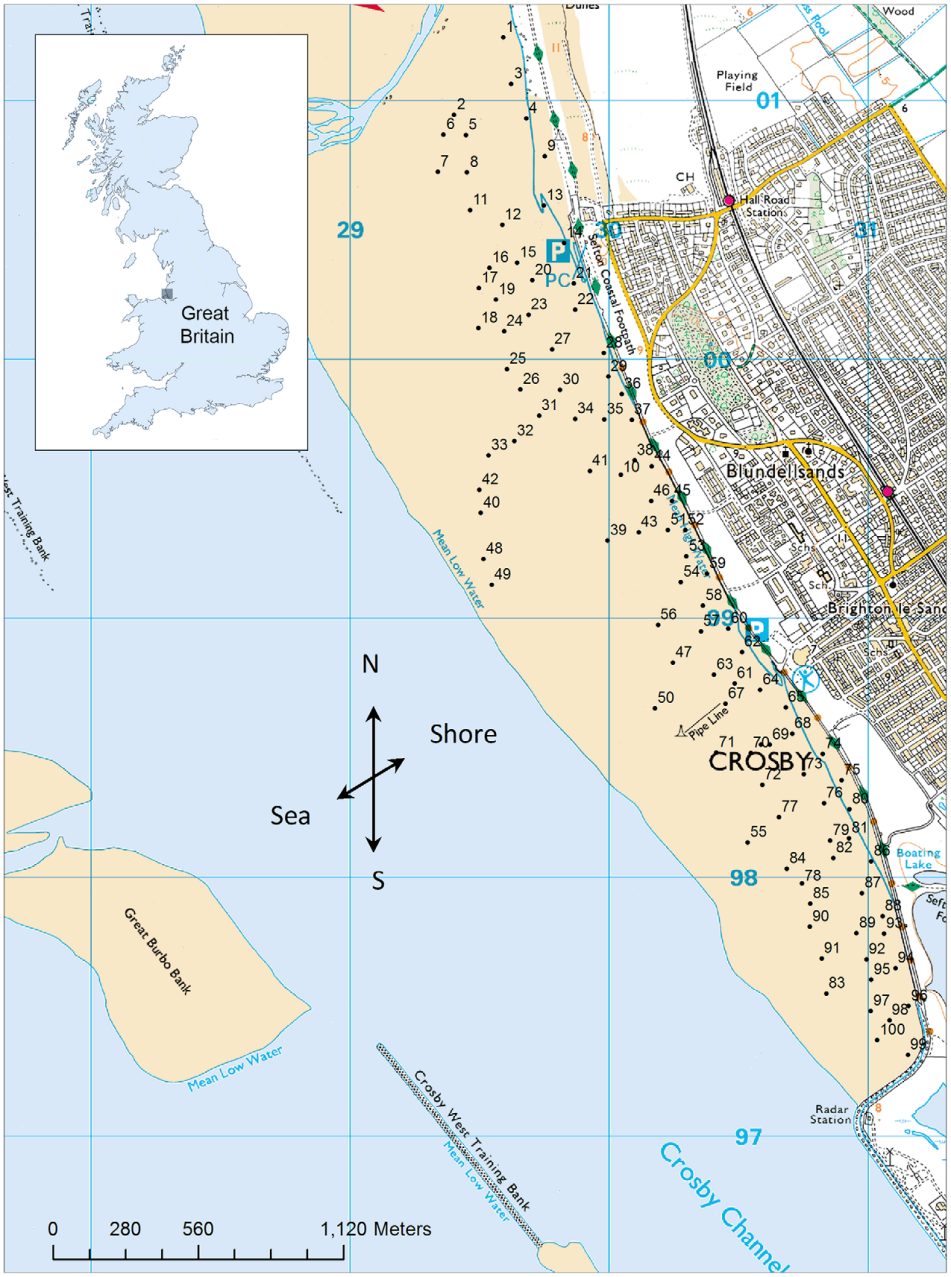
Location of the 100 Antony Gormley statues (numbered) at Crosby Beach, Liverpool. Locations of the statues are shown in relation to Great Britain. Also showing the direction of sampling positions used to assess barnacle abundance on the statues (north, south, sea, or shore) (Ordnance Survey © Crown copyright 2011).

Incorporating all potential factors that influence abundance patterns within one study is often infeasible; as such, most work has tended to focus on only one or two [30], despite evidence that it is the combination of multiple factors and the interactions between them that are often responsible for structuring resident communities of organisms [31]. Here we used variations in the distribution and abundance of an invasive intertidal acorn barnacle, *Austrominius modestus*, on a novel artificial substrate matter to develop a model that can incorporate all factors thought to be most important in determining abundance distributions of barnacle species, as well as the interactions between them. By simultaneously assessing multiple indices, using a unique field study opportunity, we aimed to gain a greater insight into the habitat requirements of *A. modestus* on a local scale and hypothesised that no one factor would be solely responsible for any patterns observed.

## Methods

### Study Site

In July 2005, 100 life-size cast-iron human figures (Fig.1) were distributed at various tidal heights along approximately 3 kilometres (km) of the foreshore at Crosby Beach, Liverpool (Fig.2) to form the art installation, ‘Another Place’, by sculptor Antony Gormley. A preliminary investigation undertaken in 2006, one year post installation, revealed the statues to be dominated by the invasive barnacle, *Austrominius modestus*, to the exclusion of all other species of barnacle (L. A. Robinson, personal observation). Before installation, the Gormley statues were blasted clean; as such they provided a pristine, hard substrate onto which organisms could settle in an otherwise sandy (unsuitable) environment. The statues are placed at a range of tidal heights, and different regions of their body differ in exposure (whether or not they are sheltered by another body part – e.g. the inner thigh versus the outer thigh; we do not treat exposure as a direct measure of wave fetch), direction (north, south, sea, and shore), and rugosity (the influence of body contours, where an area such as the groin might be more complex than the torso) (Fig.1, Table 1). As a contemporary art installation, the statues were never intended as an ecological study; however they present a unique opportunity to investigate the influence of these factors, and their interactions, on the distribution and abundance of *A. modestus* in the area.

**Table 1:** Description of the 14 sampling positions, and their associated variables, that were sampled for coverage of the barnacle *Austrominius modestus* on each of the 100 statues at Crosby Beach, Liverpool.

Sampling position	Rugosity	Height from base of feet (cm)	Direction	Exposure
Head front	1.62	180	Sea	Exposed
Head back	1.51	180	Shore	Exposed
Torso front	1.46	135	Sea	Exposed
Torso back	1.44	135	Shore	Exposed
Groin	1.70	97	Sea	Exposed
Buttocks	1.47	94	Shore	Exposed
Lower leg front	1.43	61	Sea	Exposed
Lower leg back	1.47	65	Shore	Exposed
Inner thigh left	1.42	71	North	Sheltered
Inner thigh right	1.42	71	South	Sheltered
Outer thigh left	1.51	71	South	Exposed
Outer thigh right	1.51	71	North	Exposed
Under arm left	1.45	134	South	Sheltered
Under arm right	1.45	134	North	Sheltered

Under the index of rugosity, higher values indicate a greater level of complexity with 1 being a flat surface; direction describes the direction in which each sampling position faced: sea, shore, north and south; and exposure was assessed according to whether or not the sampling position was sheltered by another body part.

### Study Species

*Austrominius modestus*, previously known as *Elminius modestus*, originates from Australasia, and whilst it was once considered to be a strictly southern genus [32], it has become commonplace and abundant in many European estuaries and other sheltered marine areas [33], [34]. In its native habitat, *A. modestus* is a prominent fouling organism of harbours and estuaries, capable of growing ‘on any sort of substratum’ [35] in the upper limit of tidal ranges [32]. In the UK and Ireland, the species occupies a similar environmental niche, being predominantly found in sheltered areas of varying salinity [27], [36]. However, in comparison to its native range, *A. modestus* is not limited to upper tidal limits and occupies a wide range of tidal heights, being predominant in the low-mid region in some locations [37]. *A. modestus* appears to occupy a similar niche as the native barnacles *Balanus balanoides* and *Semibalanus balanoides*, however its superior tolerance of fluctuating salinity and ability to reproduce all year round has enabled *A. modestus* to occupy a greater intertidal range, outcompeting these species in numerous sheltered estuarine habitats [33], [36]–[38]. In the Mersey Estuary, *A. modestus* is common and abundant on other intertidal hard substrates in the estuary near the study site (most of which are artificial) [39], but both *B. balanoides* and *S. balanoides* also occur (M. Spencer, personal observation). *A. modestus* and *Balanus* spp. (including *B. crenatus* and *B. improvisus*) have been recorded on nearby subtidal artificial substrates in the Liverpool docks [40]–[42].

**Figure 3:**
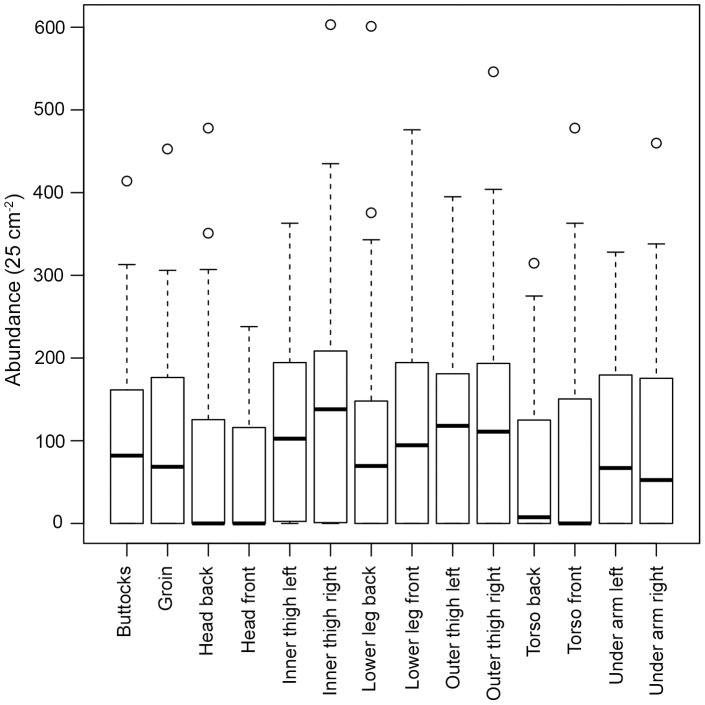
Mean total abundance of barnacles (25 cm^−2^) per sampling position **on each of the 100 statues.** In total 14 sampling positions were chosen to represent a range of environmental conditions experienced by the statues and were located at the same points on each of the 100 statues sampled at Crosby Beach, Liverpool (for statue locations see Fig. 2). Boxplots show the medians (thicker black line) and upper and lower quartiles of abundance values at each sampling point, with whiskers extending to the extremes of data points not considered to be outliers.

**Figure 4:**
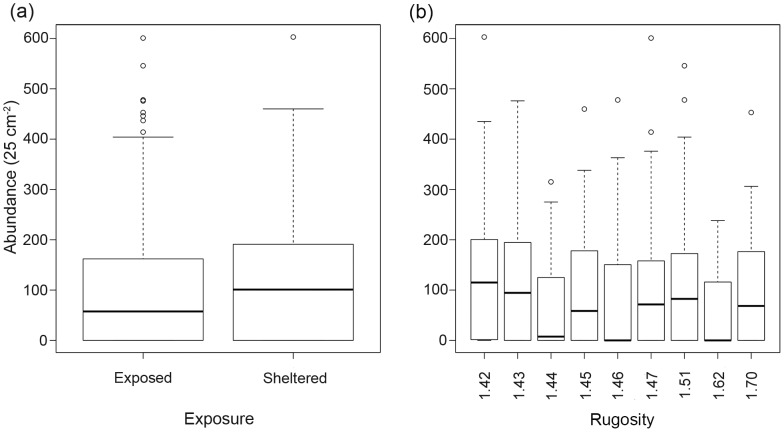
Total abundance of barnacles (25 cm^−2^) in relation to (a) exposure and (b) rugosity. Exposure was defined as whether or not the sampling position was sheltered by another sampling position and a rugosity index was used to describe the complexity of each sampling position, with higher numbers indicating greater complexity. Boxplots show the medians (thicker black line) and upper and lower quartiles of abundance values for both indices, with whiskers extending to the extremes of data points not considered to be outliers.

### Data Collection

The abundance of *Austrominius modestus* was quantified using 10×10 cm quadrats placed at 14 positions on each of the 100 statues (Fig. 2) during August 2006, one year after their installation following permission granted by Sefton Council. Positions were chosen to represent a range of heights, rugosity, and exposure, and were placed in the same location for each statue (Table 1). Quadrats, which were constructed from garden wire to allow flexibility, were subdivided into 5×5 cm sections and the numbers of individuals present in the upper left hand corner of each quadrat were counted. Rugosity ranged from the highly contoured face and groin to the relatively uniform torso. This range in rugosity was assessed by using a piece of string to measure the distance from one side of the 10×10 cm quadrat to the other, to allow the extra distance of cracks and crevices to be incorporated. The ratio of string length to quadrat width was then used as a rugosity index, with higher values indicating a greater level of complexity and a value of 1 being a flat surface. The sampling positions faced four different directions; sea, shore, north and south, and exposure was assessed according to whether or not the sampling position was sheltered by another body part.

Each statue measured 191 cm from the base of the feet to the top of the head, and sampling positions varied from between 61 and 180 cm from the base of the feet. The shore height of each sampling position on each statue was calculated as the sum of the distance from the base of the feet and the shore height of the base of the feet (estimated from the time at which the tide reached the feet of the statues using tidal curves for Liverpool). Shore heights of the statues ranged from just over 1 m to 10 m above chart datum and the heights of the sampling positions ranged from 2 m to almost 12 m. Many of the shore heights were replicated by two or more statues. The latitude and longitude of each statue was obtained using a Garmin etrex© Global Positioning System.

**Table 2:** Generalized linear mixed models (GLMMs) of barnacle abundance (numbers 25 cm^−2^) in relation to important environmental variables at Crosby Beach, Liverpool.

GLMM (Fixed effects)		d.f	*l*	AIC	Δ AIC
(a)	Location	4	−3334	6676	482
(b)	Exposure	4	−3332	6672	478
(c)	Direction	6	−3298	6608	414
(d)	Shore Height	4	−3164	6335	141
(e)	Rugosity	4	−3336	6679	485
(f)	Location+ Exposure + Direction + Shore height + Rugosity	10	−3140	6300	106
(g)	**(f) + Shore height:(Location + Direction)**	**14**	**−3083**	**6194**	**0**

Model selection was based on Akaike’s Information Criterion (AIC) with additional interactions being kept if they reduced AIC by >2. The most parsimonious adequate model on this basis is shown in bold. *n* = 1400 quadrats; d.f., degrees of freedom; *l*, log-likelihood; Δ AIC, the difference in AIC from that of the most parsimonious adequate model.

**Figure 5:**
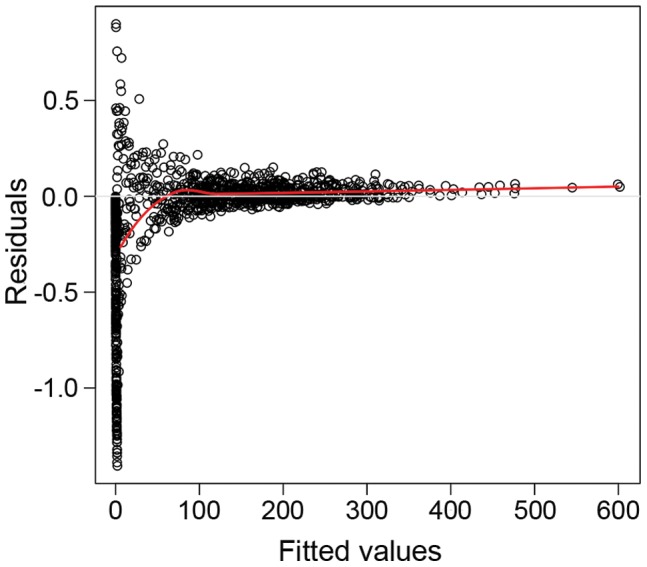
Goodness of final model fit **used to assess barnacle abundance on the statues at Crosby.** Goodness of fit of the final generalized linear mixed model is illustrated through assessment of the fitted values of the selected final model against the residuals of the model (red line indicates loess smoother).

### Statistical Analysis

Generalized linear mixed models (GLMMs) were used to investigate the effects of location, shore height, exposure, direction, and rugosity, and some of their interactions on recruitment of *Austrominius modestus* to the statues. GLMMs are useful as they can be applied to non-normal data that includes a mix of both fixed and random effects whilst also allowing for co-variation among samples [43]. We assumed that *A. modestus* counts followed a Poisson log-normal distribution, conditional on the values of the explanatory variables. The Poisson log-normal is one of several possible models for over-dispersed count data, but is particularly convenient for GLMMs [44]. We treated statue as a random effect, to account for variation among statues. We also added an additional random effect of observation, to account for unexplained variation within statues. Location (the first principal component of latitude and longitude), shore height, exposure, rugosity, and direction were treated as fixed effects. We considered models including combinations of these fixed effects and their two-way interactions (all models included both the random effects). However, due to the physical layout of sampling positions on the statues, some of these interactions (direction and rugosity, exposure and direction) were not identifiable, in the sense that models with different parameter values could fit the data equally well. We excluded any model containing such interactions. We selected the model having the lowest Akaike’s Information Criterion (AIC) among this subset of possible models.

**Table 3:** Parameter estimates for the final generalized linear mixed model (GLMM) assessing the influence of various factors on the abundance of barnacles.

Fixed effects	Estimate	Std error
(Intercept)	13.358	1.198
Location	4.482	0.606
Exposure(Sheltered)	0.339	0.138
Direction(Sea)	1.765	0.426
Direction(Shore)	0.979	0.420
Direction(South)	0.188	0.422
Rugosity	0.121	0.067
Shore height	−1.795	0.118
Location:Shore height	−0.592	0.071
Direction(Sea):Shore height	−0.360	0.067
Direction(Shore):Shore height	−0.192	0.065
Direction(South):Shore height	−0.017	0.067

**Figure 6:**
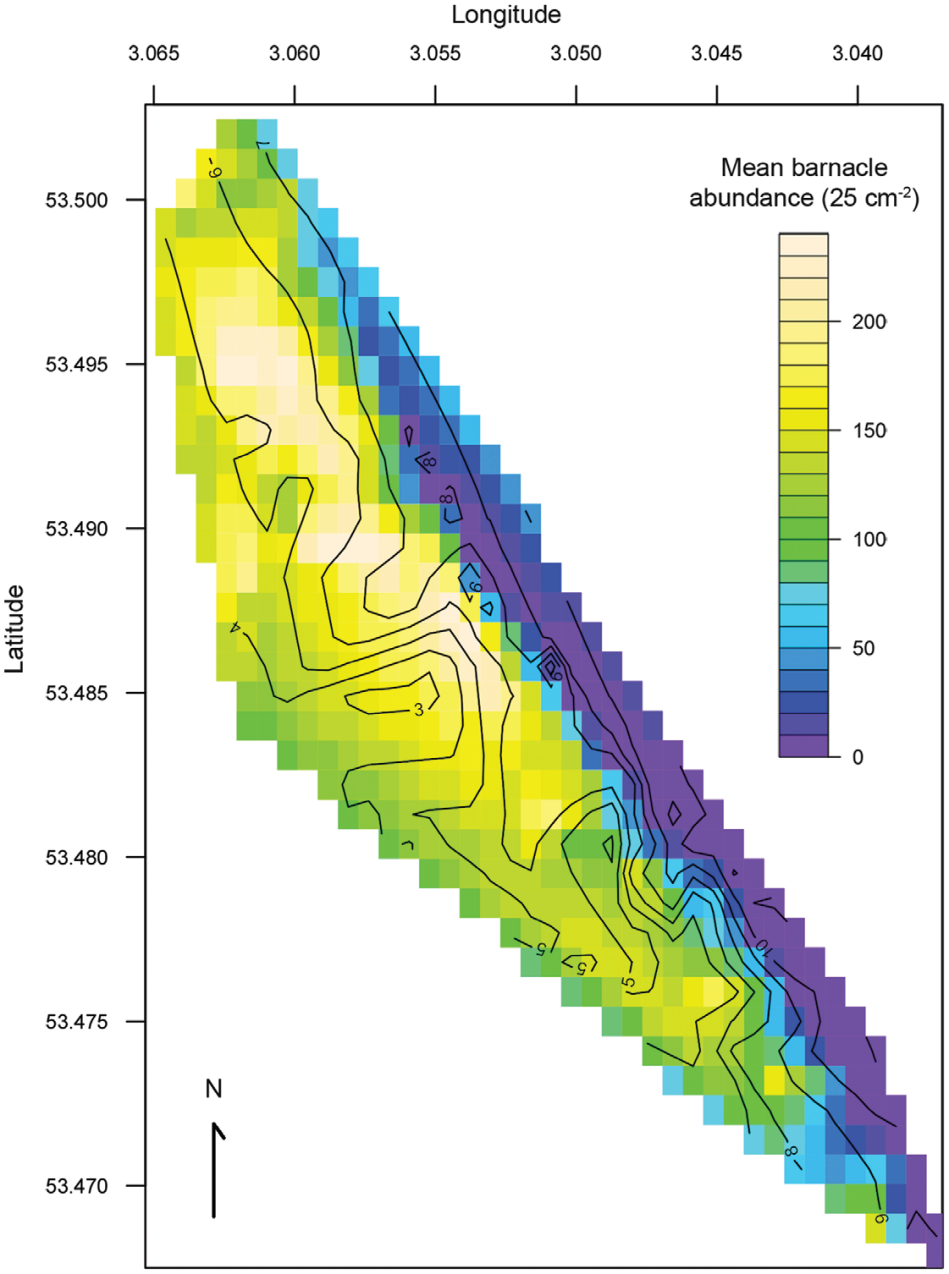
Predicted mean abundances of barnacles (25 cm^−2^) per statue position **in relation to their location.** Predicted mean abundances are based on predicted values from a generalized linear mixed model output, with contours indicating actual shore height of the statues.

It is likely that there is spatial autocorrelation among sampling positions within a statue, because positions close together in space may experience similar environmental conditions. However, because the same set of positions is sampled on every statue, these patterns can be captured by the fixed effects in the model. It is also likely that if there are many individuals at a given position on a statue, other positions on the same statues will also have many individuals, due to similarities in environmental conditions. Such patterns will be captured by the random effect of statue. The absence of post-settlement dispersal of barnacles means that we do not expect spatial autocorrelation arising from the direct influence of numbers at one position on numbers at other positions. We checked the model assumptions by visual inspection of residuals. All statistical analysis was performed using the R software package “lme4” version 0.999375-40 [45].

**Figure 7:**
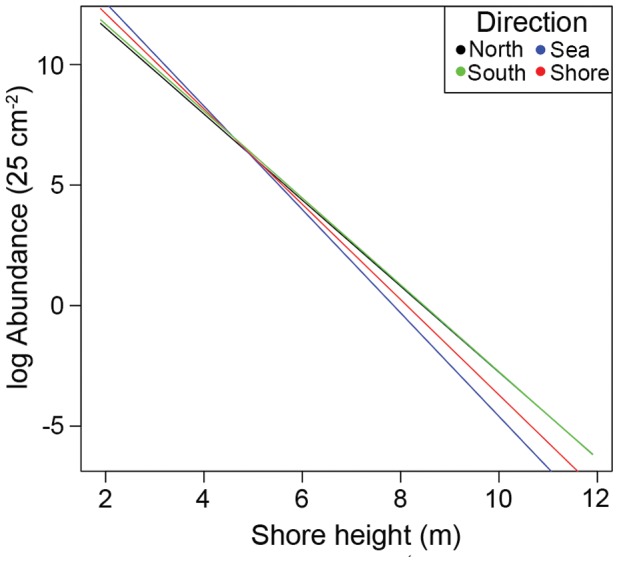
Predicted changes in log barnacle abundance (25 cm^−2^) in relation to shore height and direction. Sampling positions faced four different directions: north, south, sea, shore. Values are based on those predicted from a generalized linear mixed model output using the median value of Location (first principal component of latitude and longitude), the mean level of Rugosity of the sampling positions and exposed or sheltered levels of Exposure.

## Results

Substantial variations in the abundance of *Austrominius modestus* were observed over the sampling positions (*n* = 1400) analysed in the study (Fig. 3). The overall mean abundance (± SE) was 96±3 individuals 25 cm^−2^ with a maximum abundance of 603 individuals 25 cm^−2^. Zero counts were numerous and observed at a range of tidal heights, although all sampling positions with a height of 9.1 m and greater had zero counts except for the inner and outer thigh of two of the statues (98 and 99). The greatest mean abundance was on the inner thigh (139±12 individuals 25 cm^−2^) and the lowest on the front and back of the head (53±8 and 66±10, respectively). However, there was no strong trend in abundance in relation to sampling position, with no one position standing out as being most suitable for *A. modestus* recruitment. There did appear to be a difference between the numbers of barnacles in relation to exposure, with greater mean abundances observed on sampling positions described as sheltered (55±2 individuals 25 cm^−2^) than on those described as exposed (45±1 individuals 25 cm^−2^) (Fig.4a). There seemed to be no preference for more topographically complex sampling positions over less complex ones (Fig.4b).

The most parsimonious adequate generalized linear mixed model (GLMM) was chosen from a series of GLMMs based on its complexity and AIC value (Table 2). The final GLMM was used to understand how interactions between the various fixed effects could be affecting the distribution of barnacles on the statues. Visual inspections of the residuals indicated zero inflation, with a sharp downward trend in the smoothed residuals at low fitted values (Fig. 5 red line). Whilst the model was adequate at predicting large fitted values, smaller fitted values were more variable and there was an excess of zero counts.

The selected model included two different interactions between fixed effects that could help explain the distribution of barnacles observed on the statues (Table 3). The model showed an interaction between the locations of the sampling position along the beach and shore height. The value of location (the principal component of latitude and longitude) increases towards the southerly end of the beach. The interaction between these two fixed effects indicated a more negative effect of increasing shore height on the abundance of barnacles on sampling positions located towards the southerly end of the beach (Fig. 6). Clear differences in mean abundances and mean shore heights were observed when statues were compared based on their location: northern (statues 1–49) or southern (statues 50–100). Those statues located at the northern end of the beach were characterised by higher mean abundances and lower mean shore heights (2000±147 individuals 25 cm^−2^ at mean shore height of 4.7±0.2 m) when compared to those at the southern end (739±123 individuals 25 cm^−2^ at a mean shore height of 7.2±0.3 m).

The highest (mean) abundances (>200 individuals 25 cm^−2^) were predicted at the northerly end of the beach in the mid tidal height region, and the lowest (<50 individuals 25 cm^−2^) in most of the high tidal height region, particularly towards the southerly end (Fig. 6). The second interaction observed was between the direction of the sampling position and shore height. Increasing shore height was predicted to decrease the abundance of barnacles in all directions but was most acute on seaward and shoreward facing positions with a lesser effect observed for north and south facing positions, where abundances decreased at an almost identical rate (Fig. 7, see Fig. 2 for actual positions of statues).

## Discussion

Within a year, the invasive barnacle *Austrominius modestus* was found to have thrived on the Antony Gormley statues along the sandy beach in Crosby, Liverpool (individual abundances being in their hundreds per 25 cm^−2^ in suitable positions), opportunistically colonising and dominating the man-made installation in an environment that is otherwise void of settlement sites. Using a generalized linear mixed model approach, we successfully modelled the distribution of *A. modestus* across these statues, with high abundances predicted with greater precision. We found that while individual parameters, such as rugosity, had some influence on barnacle distribution, they were less important than the interactions between shore height and direction of the sampling position, and specific location on the beach. In these conditions, *A. modestus* was only really affected by position on the statue itself (and thus ‘design’) at the extremes of shore height tolerated. In other words, having ‘another place’ offering suitable substrate within its tidal range was enough to lead to widespread colonisation and dominance.

There is increasing concern that artificial structures could aid the spread of non-native species [23]. It is clear from this study that the addition of suitable substrate to a sheltered estuarine environment, conditions in which *A. modestus* is commonly found [34], [37], has facilitated the establishment of this species to the area. *A. modestus* has a longer reproductive period than any other barnacle in British waters [46] and can breed almost all year round. This particular physiological trait could have given it a competitive advantage over other barnacle species when colonising the statues initially, particularly when considering peak settlement in this species has been observed in the summer and autumn months [37], [47], around the time the statues were installed. However, a more recent study has found that *A. modestus* still dominates the statues, to the exclusion of all other barnacle species, and that it rapidly re-colonises areas of the statues that become available through disturbance [48].

The influence of shore height on the distribution of *A. modestus* in this area is unsurprising given the well-described zonation patterns observed in barnacle species [49]. The negative effects of shore height on abundances were most acute at the southerly end of the beach. This was most likely a result of the comparative narrowness of the beach in this region and the greater mean shore height of the statues (see Fig. 2 for statue locations). In its native range, *A. modestus* is distributed in the upper tidal region [32], [35]. In the UK, however, the species occupies a much wider range of tidal heights [38], and its distribution appears to be highly variable dependent on the relative abundances and distribution of co-occurring native species, primarily; *Cthamalus montagui, Cthamalus stellatus, Semibalanus balanoides, Balanus balanoides and Balanus crenatus* [37]. The species appears to occupy a similar ecological niche as both *B. balanoides* and *S. balanoides*, and its competitive superiority has allowed it to become dominant over these species in numerous areas [33], [37], [38]. In the absence of any competitors or predators at Crosby Beach, *A. modestus* colonised a wide range of tidal heights, only really being absent at sampling positions greater than nine metres above chart datum. Given that the spring tidal range in Liverpool Bay (in excess of 10 m), is one of the largest in the world [50], this suggests that tidal height is of little constraint for this species where other conditions are favourable.

Numbers of *A. modestus* on north and south facing positions were least affected by increasing shore height. These positions made up six of the 14 sampling positions assessed on each statue, four of which were the only positions to be described as sheltered by another body part (inner thigh and under arm, see Table 1 for full descriptions). *A. modestus* is known to recruit preferentially to sheltered shores and is often only sparsely distributed on shores that experience greater levels of exposure [27], [37]. Even though Crosby Beach itself would be described as sheltered, the added protection provided by other body parts may have promoted survival of individuals as shore height increased. An additional factor that could have influenced this relationship is the direction of prevailing currents in this area, as the transport of propagules to settlement sites is greatly dependent on coastal currents and oceanographic processes on large and small scales [51], [52].

The final model used in our study included rugosity as a fixed effect, indicating that rugosity did have some bearing on barnacle abundance. However, it was not as important as the interactions of other factors, and no perceivable effect was observed. Variations in substrate rugosity and its interaction with other environmental parameters influences the spatial distribution of barnacles, as the provision of refuges can promote post-settlement survival [31], [53], although this effect is not always observed (see [54]). The results of this study suggested that the distribution of *A. modestus* at this study site were not greatly regulated by habitat complexity. Previous studies indicate that the influence of cracks and crevices on settlement in *A. modestus* may be greater on smaller scales of approximately 1 cm [55], [56], and it is possible that the scale of rugosity used in this study was too great to generate an observable response. Alternatively, except for increasing shore height, there may be little need for refuge at this study site as wave exposure is limited and no evidence of potential predators was observed; two factors known to influence the importance of rugosity on distribution of barnacles [57], [58].

The effect of individual statue location on abundance was partly explained by the interaction with shore height variation along the beach, but the locational effect may also be dependent on local current patterns within the beach and/or proximity to seeding sites (both factors we were unable to test in this study). Whilst the current patterns in Liverpool Bay have been studied extensively [50], at present no near shore oceanographic data exist on such a small scale for the area around Crosby Beach. The statues themselves could also potentially alter water flow speeds and current patterns, as has been observed for other artificial structures [59] encouraging or discouraging recruitment to particular areas. A multi-disciplinary study integrating information on very local (within beach), alongshore and regional hydrography, with information on the distribution and genetic profiles of individuals of *A. modestus* within and at sites beyond this beach, would help to further our understanding of the ecological connectivity of this species, and how artificial structures contribute to the spread of invasive species.

Identifying the variables that influence patterns of abundance in benthic invertebrates is complex, as numerous factors and interactions between them may be responsible at both the recruitment and post recruitment stage [30]. However, if we are to understand the factors responsible for the establishment and spread of invasive species, so that predictions of invasion success to new habitats can be made, a sound knowledge of the variables that determine population success is essential [60]. In this study, we have outlined the environmental parameters that influence the patterns of abundance observed in the invasive barnacle *Austrominius modestus* on an artificial substrate in an area of otherwise unsuitable habitat. This species has already displaced native barnacle species in some regions of the UK [37] and its ability to colonise and survive in large numbers in environments inhospitable to other species, combined with its rapid rate of spread, are a cause for concern [33]. With studies predicting a positive association between *A. modestus* recruitment and milder winter conditions [61], in addition to an ever-increasing presence of artificial structures in marine environments, the continued spread and dominance of this species can be expected.
